# Identification and whole-genome sequencing analysis of *Vibrio vulnificus* strains causing pearl gentian grouper disease in China

**DOI:** 10.1186/s12866-022-02610-1

**Published:** 2022-08-16

**Authors:** Zun Wu, Yating Wu, Haofeng Gao, Xuexin He, Qiang Yao, Zhanglei Yang, Jinyi Zhou, Linting Ji, Jinwei Gao, Xuying Jia, Yong Dou, Xiaoyu Wang, Peng Shao

**Affiliations:** 1 Tianjin Key Lab of Aqua-Ecology and Aquaculture, College of Fisheries, Tianjin Agricultural University, Tianjin, 300384 People’s Republic of China; 2 Tianjin Fisheries Research Institute, 422 Jiefang Nan Road, He Xi District, Tianjin, 300221 People’s Republic of China

**Keywords:** *Vibrio vulnificus*, Pearl gentian grouper, Virulence genes, Antimicrobial susceptibility, Whole-genome sequencing

## Abstract

*Vibrio vulnificus* is a pathogenic bacterium that causes disease in marine fish, affecting fish farming and human health worldwide. In May 2021, in the Bohai Bay region, a disease broke out in commercially farmed pearl gentian grouper (♀*Epinephelus fuscoguttatus* × ♂*Epinephelus lanceolatus*), causing huge economic losses. The diseased fish had skin lesions, water accumulation in their abdomens, and showed tissue and organ damage. *V. vulnificus* biotype 2 has been reported in eels and other marine fish, but it is less reported in pearl gentian grouper. In this study, the pathogenic strain isolated from diseased fish was identified as *V. vulnificus* EPL 0201 biotype 2 on the basis of physiological and biochemical characteristics and the results of 16S rRNA gene and *gyrB* sequencing, virulence gene detection, and recursive infection experiments. To gain a comprehensive understanding of the pathogenicity and drug resistance of this strain, whole-genome sequencing was performed. Whole-genome analysis showed that the gene map of this strain was complete. The Virulence Factor Database annotation results showed that this strain had the key virulence factor genes *vvhA* and *rtxA*, which cause host disease. In addition, this strain had genes conferring resistance against cephalosporins, aminoglycosides, tetracyclines, and sulfonamides. Antimicrobial susceptibility testing confirmed the presence of these resistance genes identified in the genome. The results of this study show that *V. vulnificus* EPL 0201 biotype 2 is a multi-drug resistant strain with high pathogenicity.

**Supplementary Information:**

The online version contains supplementary material available at 10.1186/s12866-022-02610-1.

## Introduction

*Vibrio* disease, also known as pathogenic hemorrhagic sepsis, is a bacterial disease caused by pathogenic *Vibrio* species, which affects a variety of aquatic animals, including fish, shellfish, shrimp, and crabs [1]. *V. vulnificus* belongs to the genus *Vibrio* and is a gram-negative, rod-shaped, halophilic opportunistic pathogen. It was first identified as halophilic *Vibrio* in 1976 [2] and was named *V. vulnificus* in 1979 [3]. *V. vulnificus* is an important pathogen of human beings and aquatic animals, and can be divided into three biotypes. *V. vulnificus* biotype 1 represents the first isolated subtype, which mainly infects humans [4]. The route of infection can be through direct contact with seawater, wounds, and the consumption of aquatic products, such as raw shellfish, carrying *V. vulnificus* [5, 6]. *V. vulnificus* infection can cause sepsis and seriously endanger human health [7]. This infection has also been reported in fish [8]. *V. vulnificus* biotype 2 was first identified in European eels (*Anguilla anguilla*) and was isolated from infected eels [9]. Diseases caused by *V. vulnificus* biotype 2 affect eels and other mariculture fish, especially in fish farms. In recent years, there have been many reports of diseases caused by *V. vulnificus* in marine fish, including grouper (*Epinephelus coioides*), half-smooth tongue sole (*Cynoglossus semilaevis*), large yellow croaker (*Pseudosciaena crocea*), mullet goby (*Mugilogobius chulae*), and gilthead seabream (*Sparus aurata*) [10–14]. Symptoms include skin lesions, and in severe cases, the disease may cause ulcers, abdominal effusion, enteritis, and tissue and organ lesions. The disease has a rapid onset and causes high mortality. Some researchers have suggested that all strains of *V. vulnificus* biotype 2 be considered pathogens with a special ability to cause *Vibrio* infections in fish, suggesting the name “*Piscis*” for this biotype [15]. *V. vulnificus* biotype 3 has only been reported in Israel and causes wound infection and bacteremia in humans [16].

In May 2021, a disease broke out in a pearl gentian grouper breeding pond in a marine farm in the Bohai Bay area and caused serious economic losses. The laboratory obtained dominant bacteria from the diseased pearl gentian grouper and named it EPL 0201. In this study, we isolated the pathogen; performed bioinformatics analysis, virulence gene testing, API 20 Bacterial Identification System analysis, recursive infection experiments, and whole-genome sequencing; and tested antimicrobial drug susceptibility to investigate the pathogenic and resistance mechanisms of the bacterium. Our findings provide the basis for further research on the pathogenicity of this bacterium and the development of strategies to prevent the spread of infection.

## Material and methods

### Diseased fish

Diseased and moribund pearl gentian groupers (body length 15–25 cm; body weight 50–150 g) were collected from a seawater factory farm in the Binhai New district, Tianjin City, China, where the motility of the cultured pearl gentian grouper was > 75%. The diseased fish were transferred to the Tianjin Agricultural University Key Lab of Aqua-Ecology and Aquaculture, and after anesthetization using MS-222 (Sigma-Aldrich, St. Louis, MO, USA), a camera (Nikon, Tokyo, Japan) was used to record their symptoms.

### Isolation and identification of bacteria

Under sterile conditions, the gills, surface mucus, and diseased tissue of the diseased fish were observed under a microscope by wet mount, and no fungi and parasites were found. Subsequently, tissues from the spleen, kidney, and intestine of the diseased fish were taken under sterile conditions, pulverized with sterile PBS buffer, and then streaked on BHI fixed medium plates (Landbridge, Beijing, China), followed by culturing at 28 °C for 24 h. Dominant uniform bacterial colonies were observed on the culture plates after incubation. A single dominant bacterial colony was selected and inoculated on the same medium to obtain a pure isolate. The pure isolate thus obtained was tentatively named EPL 0201 was stored at − 80 °C in BHI broth containing sterile glycerol at a final concentration of 15% (v/v). This bacterial isolate was gram stained, and its morphology was observed using a light microscope (Leica, Wetzlar, Germany). The isolates were fixed in 2.5% electron microscope fixative solution for 2 h in the dark, following which the morphology was observed using a transmission electron microscope (Hitachi, Tokyo, Japan). Molecular identification of the isolate was performed via 16S rRNA gene and gyrase B (*gyrB*) sequencing [17, 18]. The 16S rRNA gene and *gyrB* amplification products were sent to Sangon Biotech (Shanghai, China) for sequencing. Nucleotide sequences were submitted to GenBank (https://www.ncbi.nlm.nih.gov/genbank/) [19]. Similarity analysis of the 16S rRNA gene and *gyrB* sequences was performed using the EzBioCloud database (https://www.ezbiocloud.net/) [20]. MEGA 7.0 software (https://www.megasoftware/) was used for phylogenetic analysis based on individual and concatenated sequences [21]. Distances and clustering were estimated using the neighbor-joining method (bootstrap = 1000).

### Testing for virulence genes

The presence of some virulence genes, including *vcgC*, *vvhA*, rtxA, *Wza*, and *ompU*, was detected by performing PCR with their respective specific primers listed in Supplementary Table F1 [22–24].

*vcgC* is a unique virulence gene of *V. vulnificus* biotype 1 and has weak pathogenicity but is important for distinguishing between *V. vulnificus* biotypes 1 and 2 [22]. *vvhA* (hemolysin A) codes for a 51-kDa water-soluble protein, which is the only cytotoxin extracellularly secreted by *V. vulnificus*. The *V. vulnificus* strains carrying this virulence factor gene are highly pathogenic [25]. *vvhA* is associated with tissue necrosis in the small intestine, which is often followed by the spread of infection to the bloodstream and other tissues [26]. *rtxA* (cytolysin) is the most interesting virulence gene carried by *V. vulnificus* biotype 2 and is highly pathogenic [27]. After the bacteria come into contact with host cell, the gene produces a cytotoxin that causes the host cell to lyse. At present, it is believed that the pathogenesis of *V. vulnificus* infection mainly depends on the synergistic effect of multiple virulence factor genes. In addition to the well-defined main virulence genes *vvhA* and *rtxA*, other virulence genes, such as *Wza* (capsular polysaccharide outer membrane protein) and *OmpU* (conserved outer membrane porin), are thought to play a role in the pathogenic process of *V. vulnificus* [28]. *Wza* plays an important role in protecting bacteria from the host’s innate immune response and is widespread among pathogenic *Vibrio spp.*[29]. *OmpU* can mediate bacterial adhesion to host cells and enhance the pathogenicity of *V. vulnificus* [28].

### Physiological and biochemical characteristics

The physiological and biochemical characteristics of the bacterium were analyzed using the API 20E bacterial identification system [30]. The results of the physiological and biochemical characterization of the isolated strain EPL 0201 were evaluated according to Bergey’s Manual of Determinative Bacteriology [31] and Bacteria and Fungi from Fish and Other Aquatic Animals: A Practical Identification Manual [32].

### Recursive infection

For recursive infection experiments, healthy pearl gentian groupers (body length, 15–20 cm; body weight, 50–100 g) were purchased from Qianhaiyuan Farm in Tianjin, China, and kept in a 1500-L circulating water system similar to the farm environment for 2 weeks. During this period, the fish were given commercial feed (Haitong, Weifang, China) twice daily, and the water temperature was maintained at 25 °C ± 0.5 °C with 25 ppt salinity.

Before the recursive infection experiments, the fish were randomly divided into 6 groups of 12 each. Each group was placed in an 80-L water tank. Bacterial suspension (1.0 × 10^7^–1.0 × 10^3^ CFU mL^−1^) was prepared with BSMFL01 (Baso, Guangzhou, China). The fishes in each experimental group were intraperitoneally injected with 0.1 mL of the bacterial suspension, whereas those in the control group were injected with 0.1 mL of 0.85% NaCl. The experimental fish were continuously observed for 7 days after injection, during which normal feeding, water changes, infection incidence, and mortality were recorded, and the LD_50_ was calculated using the Bliss method [33].

### Whole-genome sequencing and analysis

The EPL 0201 strain was grown and harvested in the late exponential growth phase. Total genomic DNA (gDNA) was extracted using bacterial gDNA extraction kits (TaKaRa, Tokyo, Japan). The extracted DNA was detected using agarose gel electrophoresis, and the DNA quality was analyzed using IMPLEN P300 (IMPLEN, Munich, Germany). High-quality bacterial DNA was sent to BioMarker (Beijing, China) and sequenced using the PacBio RSII high-throughput sequencing platform (Pacific Biosciences, Menlo Park, CA, USA).

### Genome functional annotation

#### Public database annotations

The whole-genome sequence of the EPL 0201 strain was obtained using the GenBank non-redundant protein (NR) database [34]. Evolutionary genealogy of genes: Non-supervised Orthologous Groups (eggNOG) [35]; Gene Ontology (GO) [36]; and the Kyoto Encyclopedia of Genes and Genomes (KEGG) [37] were used to predict gene functions.

#### Proprietary database annotation

We used the Virulence Factor Database (VFDB) [38]and the Comprehensive Antibiotic Research Database (CARD) [39] to identify virulence and resistance factors.

### Antimicrobial susceptibility

Antimicrobial susceptibility testing was performed using the Kirby–Bauer paper diffusion method [40]. The bacterial suspension was evenly distributed on Mueller–Hinton agar plates using sterile cotton swabs, and antibiotic disks (Binhe Microorganism Reagent, Hangzhou, China) were placed on the surface of the culture plate using sterile tweezers. The plates were subsequently incubated at 28 °C for 24 h, following which the zone of inhibition was measured and the colonies were counted using a colony counter (Czone 8, Shineso Science & Technology Co., Ltd., Hangzhou, China). The sensitivity was determined according to the manufacturer’s instructions (Binhe Microorganism Reagent).

## Results

### Identification of isolates

**Fig. 1:**
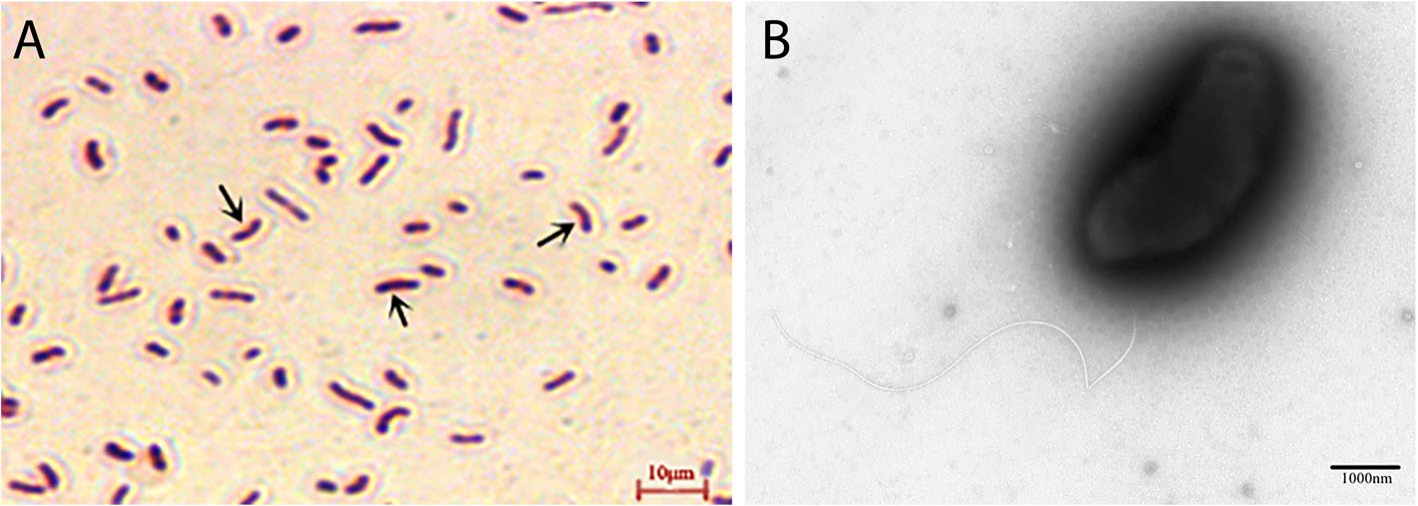
Varying morphology and flagella of strain EPL 0201 observed under light and electron microscopes. **A** Micro-curved rod-shaped bacteria (black arrow). **B** Micro-curved rod-shaped bacteria and flagella observed under an electron microscope

**Fig. 2:**
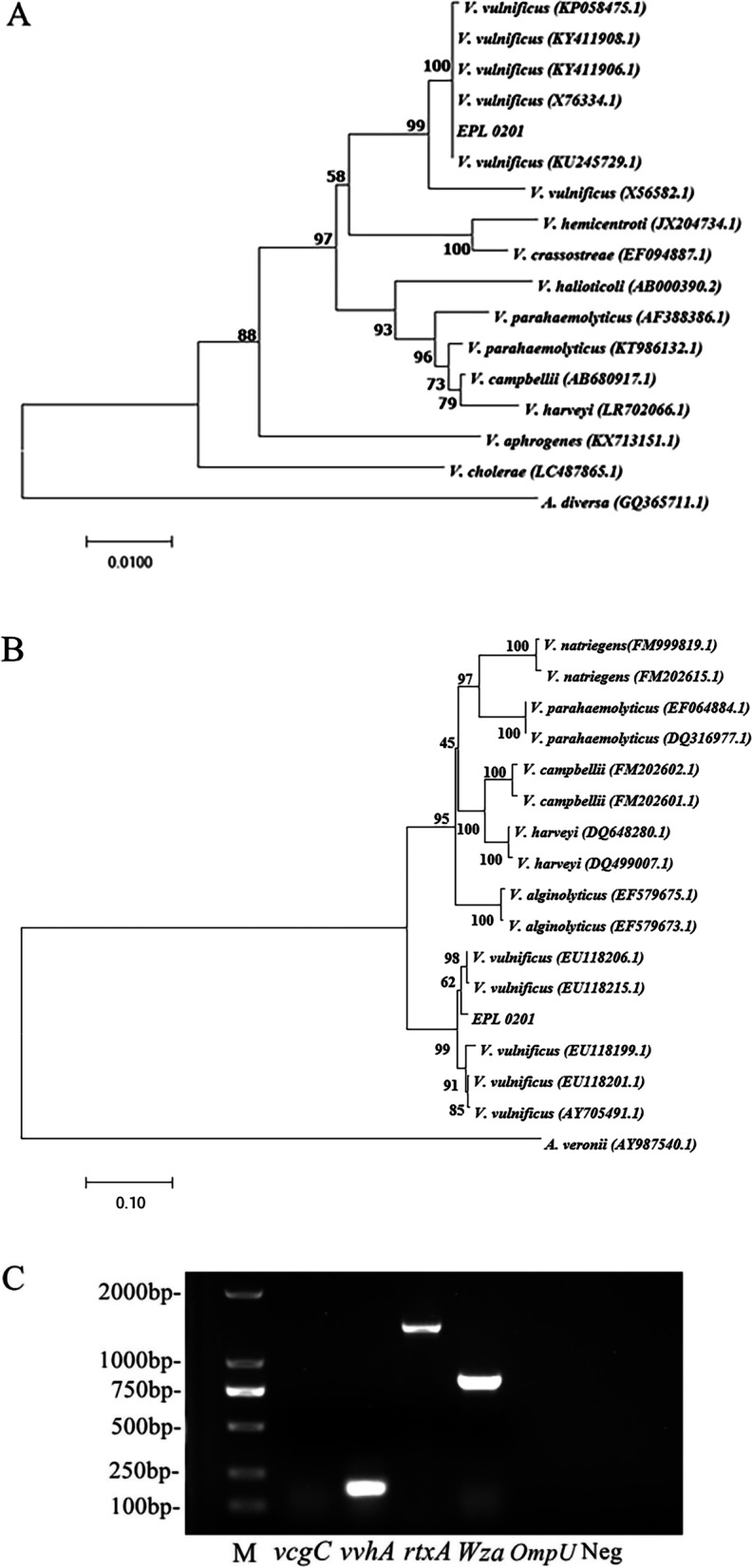
Molecular identification and virulence gene detection of strain EPL 0201. **A** Phylogenetic tree of *Vibrio* spp. based on the 16S rRNA gene sequence constructed using the neighbor-joining method. Bootstrap values were based on 1000 replicates, and percentage bootstrap values are shown at each node. **B** Phylogenetic tree of *Vibrio* spp. based on *gyrB* sequence constructed using the neighbor-joining method. Bootstrap values were based on 1000 replicates, and percentage bootstrap values are shown at each node. **C** Agarose gel electrophoresis did not show the presence of *vcgC* and *OmpU* but showed the presence of *vvhA*, *rtxA*, and *Wza* in the PCR products. M, Marker; Neg, negative control. Original agarose gel electropherogram (Supplementary Fig. S6)

Gram staining indicated that the isolated strain was a rod-shaped gram-negative bacteria (Fig. 1A). Electron microscopy revealed curved rod-shaped bacteria with micro-arc-shaped terminal single flagella (Fig. 1B). The 16S rRNA gene and *gyrB* sequences were 1434 and 1154 bp in length, respectively, and showed 100% identity with *V. vulnificus* KU245729.1 and 99.86% identity with *V. vulnificus* EU118215.1, respectively. A phylogenetic tree was constructed using MEGA 7.0 (Fig. 2A, B), and the EPL 0201 strain was found to cluster with *V. vulnificus* [19]. The virulence factor gene *vcgC* is unique to *V. vulnificus* biotype 1 and can be used to differentiate between *V. vulnificus* biotypes 1 and 2 [41]. *vvhA* is a key pathogenic gene for determining the pathogenicity of *V. vulnificus*, and 90% of pathogenic *V. vulnificus* carry this factor [27]. The agarose gel electrophoresis did not show the presence of *vcgC* and *OmpU* but showed the presence of *vvhA*, *rtxA*, and *Wza* in the PCR products (Fig. 2C). Therefore, it was preliminarily concluded that the EPL 0201 strain was *V. vulnificus* biotype 2 and had certain pathogenicity.

### Analysis of physiological and biochemical characteristics

The API 20E is a useful tool for delineating *V. vulnificus*, can identified *V. vulnificus* biotypes 1 and 2 with 85 ~ 99% accuracy [42, 43]. The main phenotypic characteristics that differentiate *V. vulnificus* biotype 1 from *V. vulnificus* biotype 2 are indole production, ornithine decarboxylation, growth at 42 °C, and acid production from mannitol [43]. The code of the EPL 0201 strain in the API 20E coding manual was 500,600,557 (Table 1). This code was consistent with the *V. vulnificus* ATCC 33,149 biotype 2 strain. Furthermore, this strain did not grow in 0, 8, or 10% NaCl peptone water but did grow in 3, 6, and 7% NaCl peptone water, which is typical of *V. vulnificus* [43]. These results were consistent with the physiological and biochemical characteristics of *V. vulnificus* biotype 2, and the strain was determined to be *V. vulnificus* biotype 2.

**Table 1:** Physiological and biochemical characteristics of pathogenic strain EPL 0201 and other *V. vulnificus* strains

Characteristics 1	EPL 0201 1	*V. vulnificus* ATCC 33,149 (biotype 2)	*V. vulnificus* ATCC27562 (biotype 1)
Gram nature	−	−	−
Hemolysis	β	β	β
Motility	+	+	+
Oxidase	+	+	+
Catalase	+	+	+
ONPG	+	+	+
Arginine dihydrolase	−	−	−
Lysine decarboxylase	+	+	+
Ornithine decarboxylase	−	−	+
Nitrate reduction	+	+	+
H_2_S production	−	−	−
Indole production	−	−	+
Urease	−	−	−
Voges–Proskauer	−	−	−
Gelatinase	+	+	+
Oxidative fermentation	F	F	F
Glucose	+	+	+
Arabinose	−	−	−
Inositol	−	−	−
Mannitol	−	−	+
Sorbitol	−	−	−
Sucrose	−	−	−
Rhamnose	−	−	−
Melibiose	−	−	−
Amygdalin	−	−	−
Growth on TCBS	G	G	G
Growth in 0% NaCl	−	−	−
Growth in 3% NaCl	+	+	+
Growth in 6% NaCl	+	+	+
Growth in 7% NaCl	+	+	+
Growth in 8% NaCl	−	−	−
Growth in 10% NaCl	−	−	−
Growth at 42℃	−	−	+
O/129 (10 μg)	S	S	S
O/129 (150 μg)	−	−	+

*Note*: + Positive, − Negative, *β* β-hemolytic, *G* Colonies grow green on TCBS, *S* Sensitive, *ONPG* Ortho-nitrophenyl-β-galactoside

### Recursive infection

**Fig. 3:**
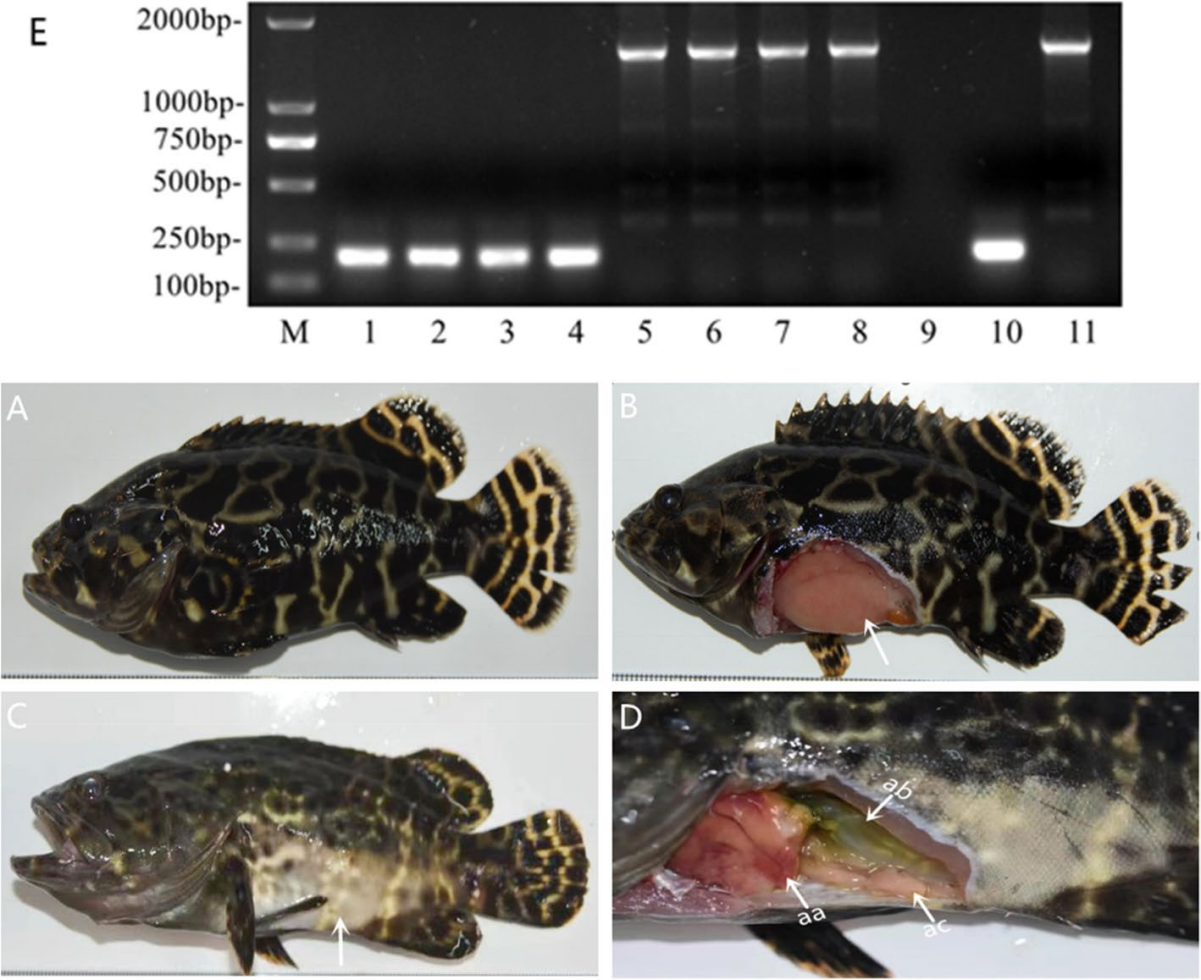
Symptoms of fish tested for recursive infection. **A** Body chart of experimental fish in control group. **B** Abdominal cavity of fish in control group (white arrow). **C** Phosphorus loss in a large area of the skin (white arrow). **D** Hemorrhagic necrosis of the liver (aa white arrow); a large amount of fluid in the abdominal cavity (ab white arrow); and intestinal inflammation (ac white arrow). **E** Agarose gel electrophoresis of PCR products of *vvhA* and *rtxA* from the suspected *V. vulnificus* isolates. M, Marker (unit, bp); 1–4, PCR products of *vvhA* from *V. vulnificus* in samples from four diseased fishes in the 1 × 10^7^–1 × 10^4^ CFU mL^−1^ groups; 5–8, PCR products of *rtxA* from *V. vulnificus* in samples from 4 diseased fishes in the 1 × 10^7^–1 × 10^4^ CFU mL.^−1^ groups; 9, negative control; 10–11, positive control (PCR products of *vvhA* and *rtxA* from EPL 0201 isolate). Original agarose gel electropherogram (Fig. S6)

The results of the recursive infection test using the *V. vulnificus* EPL 0201 strain isolated from the diseased pearl gentian groupers showed that all fishes died 7 days after the injection of the bacterial suspension at a concentration of 1 × 10^7^ CFU mL^−1^. In addition, mortality of varying degrees was observed after 7 days in the groups injected the bacterial suspension at a concentration of 1 × 10^6^–1 × 10^4^ CFU mL^−1^. The symptoms of the diseased fish in the recursive infection experiment were same as those of the naturally occurring fish [44]. The diseased fish developed skin lesions, water accumulated in their abdomens, and their tissues and organs were diseased and necrotic (Fig. 3). The fishes in the control group (0.85% NaCl) and the other experimental group (1 × 10^3^ CFU mL^−1^) did not show any symptoms and none died (Table 2). Bacteria were isolated from the spleen, kidney, and intestinal tract of the dying fish, and a large number of bacteria with the same colony morphology, size, and physiological and biochemical characteristics as the original infecting *V. vulnificus* EPL 0201 could be isolated. Four strains of *V. vulnificus* identified based on the physiological and biochemical characteristics were randomly selected for virulence gene detection, and the results showed that they all carried *vvhA* and *rtxA*. The LD_50_ calculated using the Bliss method was 1.097 × 10^5^ CFU g^−1^ (Table 2). The results of the recursive infection experiment showed that *V. vulnificus* EPL 0201 had high pathogenicity.

**Table 2:** Results of recursive infection test following intraperitoneal injection

**Group**	**No. fish**	**Bacteria administered**	**Concentration (CFU mL**^−1^**)**	**Dose (mL)**	**Mortality (%)**
1	12	*V. vulnificus*	1.0 × 10^7^	0.1	100%
2	12	*V. vulnificus*	1.0 × 10^6^	0.1	83%
3	12	*V. vulnificus*	1.0 × 10^5^	0.1	58%
4	12	*V. vulnificus*	1.0 × 10^4^	0.1	25%
5	12	*V. vulnificus*	1.0 × 10^3^	0.1	0%
6	12	0.85% NaCl (control)	** − **	0.1	0%

### Whole-genome sequencing analysis of EPL 0201

#### Sequencing data quality control information

The gDNA of *V. vulnificus* EPL 0201 was detected using 1% agarose gel electrophoresis. The four groups of bacteria-like DNA bands were complete and clear, with no tailing and no residual RNA (Supplementary Fig. S1). The DNA quality test results showed that the OD_260 nm_/OD_280 nm_ values were between 1.7 and 2.2, which were within the appropriate limit. Sequencing was performed using the PacBio RSII high-throughput sequencing platform. The raw data is shown in Supplementary Table F2, the filtered data in Supplementary Table F3, and the length distribution of the filtered data in Supplementary Fig. S2.

#### Genome assembly

**Fig. 4:**
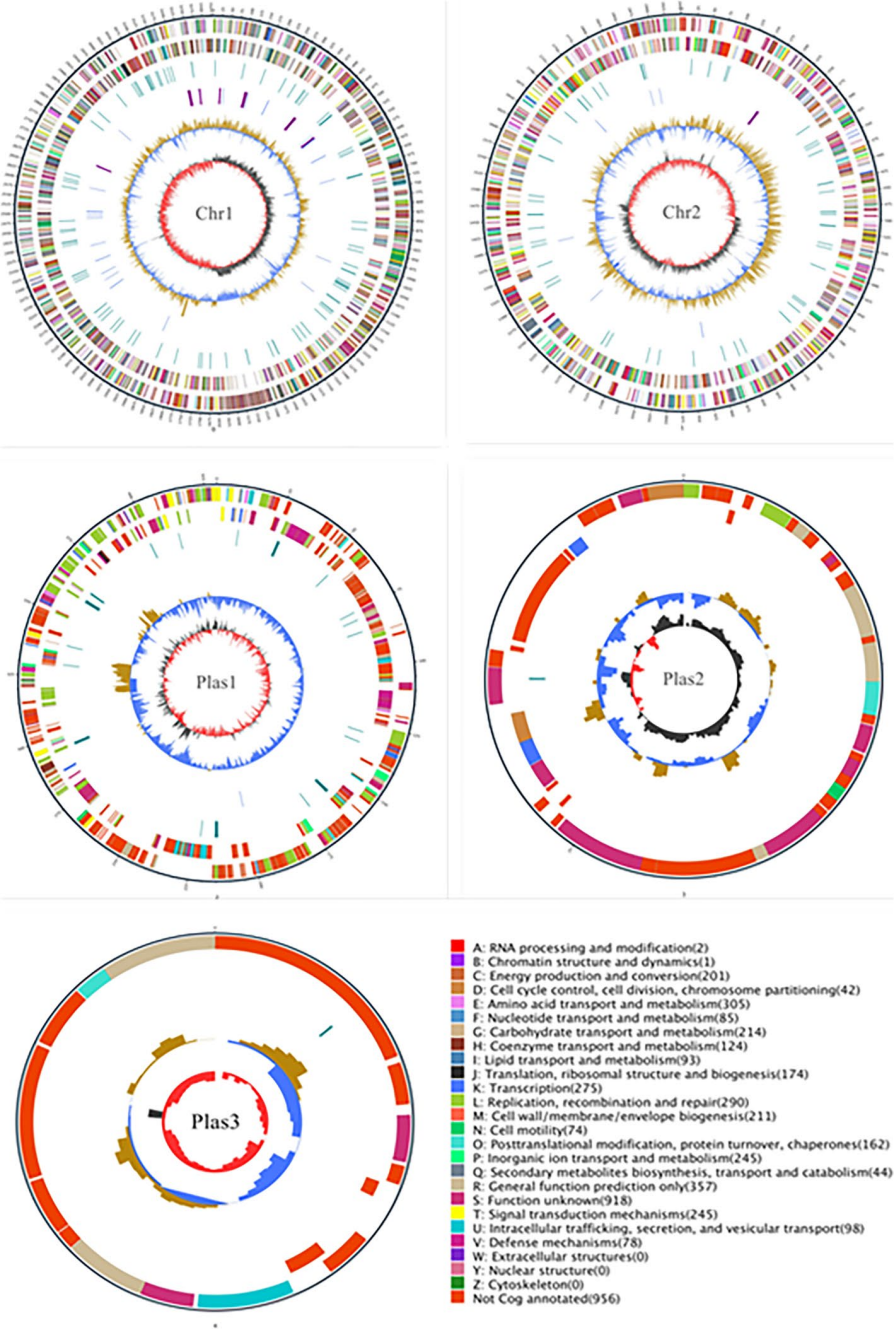
GC content and average depth correlation analysis chart. Note: The outermost circle represents the genome size, with each scale being 5 kb. The second circle and the third circle represent genes on the positive and negative strands of the genome. The different colors represent different COG functional classifications. The fourth circle represents a repetitive sequence, the fifth circle represents tRNA (blue) and rRNA (purple), and the sixth circle represents the GC content. The light yellow part indicates that the GC content of the region is higher than the average GC content of the genome, whereas the blue part indicates that the GC content of the region is lower than the average GC content of the genome. The higher the peak value, the greater the difference in GC content. The innermost circle represents GC skew, wherein the dark gray parts represent the regions where the G content is greater than the C content and the red parts represent the regions where the C content is greater than the G content

The filtered subreads data were assembled using HGAP software (Supplementary Table F4). For assembled and predicted genomic information, such as tRNA, rRNA, repeats, GC content, and gene function information, we used Circos v0.66 software to draw circular genome maps (Fig. 4) [45]. The strain was found to have three complete chromosomes (Chr1, Chr2, and Plas1) and two closed circular plasmids (Plas2 and Plas3). Circular genome map assembly metrics is presented in Supplementary Table F5.

### Functional annotation of the genome

#### NR database annotations

NR protein database is a non-redundant protein database created and maintained by the NCBI. The database includes comprehensive protein sequence and annotation information along with corresponding species information. The NR database generally enables a more comprehensive annotation of genes compared with other NCBI databases. However, one disadvantage of this database is that many protein sequences and annotations have not been verified, and the reliability needs to be improved.

**Fig. 5:**
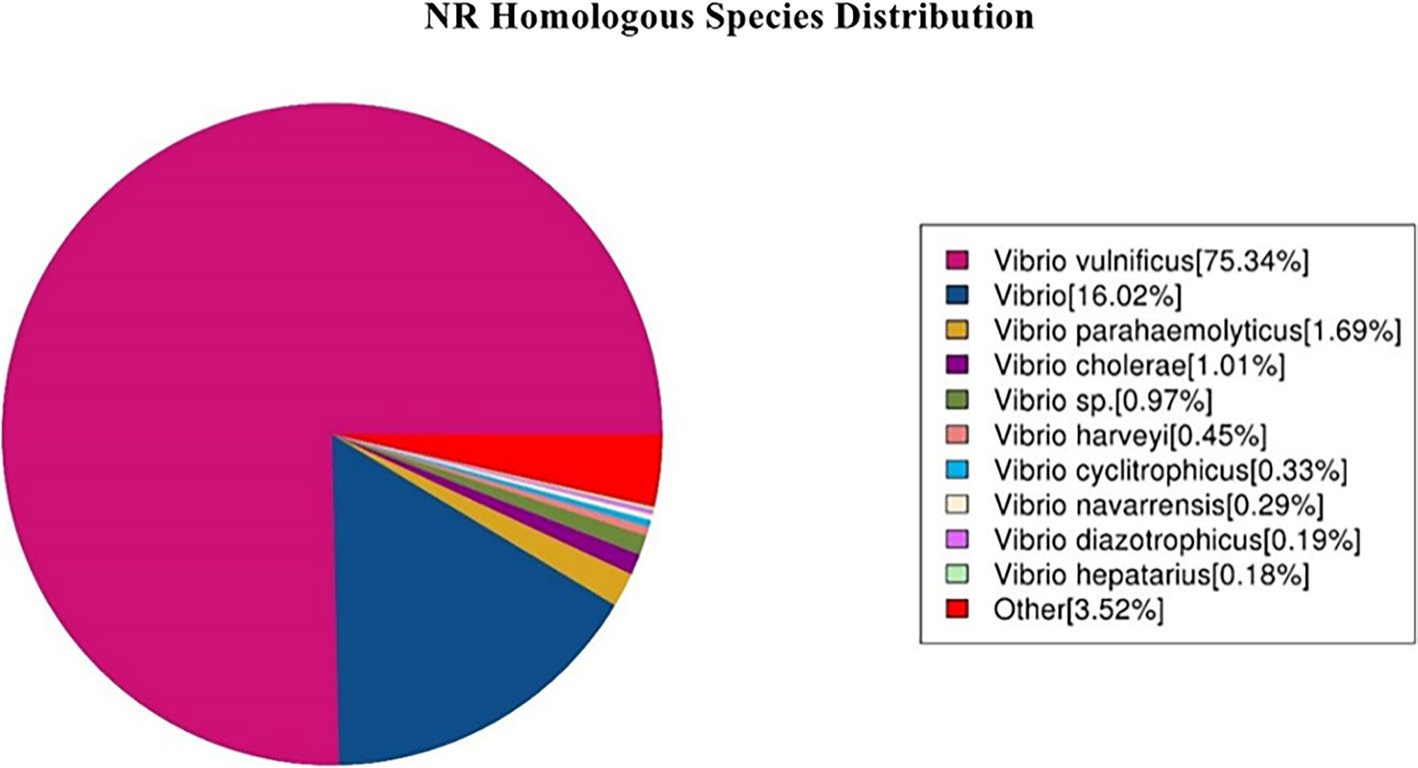
Species distribution map of the results of sequence alignment using the NR database. Note: Different colors represent different species

Annotation using the NR database revealed that *Vibrio* accounted for 96.48% of the organisms showing sequence homology with the EPL 0201 strain (Fig. 5). Further, *V. vulnificus* accounted for 75.34% and others accounted for 3.52% of the organisms showing sequence homology with the EPL 0201 strain.

#### eggNOG database annotation

The eggNOG database is a database of biological orthologous gene clusters [35]. It is continuously updated based on the COG database [46], which contains data about clusters of orthologous groups. The proteins are assumed to be derived from an ancestral protein and have the same function. The database is generated by comparing the protein sequences of complete genomes.

Using the data from eggNOG (Supplementary Fig. S3), the genome was divided into 25 categories with a total of 4000 genes. The number of S (function unknown) annotations was significantly higher than that of other groups. A (RNA processing and modification), B (chromatin structure and dynamics), W (extracellular structures), Y (nuclear structure), and Z (cytoskeleton) were the five categories with the lowest proportions.

#### GO database annotation

GO is a database established by the Gene Ontology Consortium to build a framework in which gene and protein functions are defined and described for multiple species [36]. The highest levels of categorization in the GO database are *cellular component*, which is used to describe subcellular structure, location, and macromolecular complexes; *molecular function*, which is used to describe the functions of individual gene products; and *biological process*, which is used to describe the biological process in which the gene product participates.

The GO annotations indicated that the genes were divided into 36 functional groups and summarized into three categories (Supplementary Fig. S4). The EPL 0201 strain had 4,803 annotations related to *cellular component*, 4,149 annotations related to *molecular function*, and 6,103 annotations related to *biological process*. Among the 36 functional components, the 3 categories with the highest number of genes were catalytic activity, metabolic process, and binding, whereas the 3 categories with the lowest number of genes were cell killing, developmental process, and biological adhesion.

#### KEGG database annotation

The KEGG database is a genomic information database for the systematic analysis of gene function [37]. This database integrates genomic, biochemical, and omics information and produces biological pathway maps of different types of biological processes, thereby helping researchers to study gene and expression information as a whole network.

The KEGG annotations revealed 49 biological pathway genes. The biological pathways were divided into four categories, with each category further subdivided into two levels (Supplementary Fig. S5). Ribosomes in genetic information processing, two-component systems in environmental information processing, bacterial chemotaxis in cellular processes, and biosynthesis of amino acids in metabolism were most highly represented in the pathways.

#### VFDB annotations

VFDB is a database that contains information on most of the virulence factors from bacterial pathogens that have been completely described [38]. These pathogenic factors help pathogenic bacteria establish infection in the host, respond to the host’s immune system, survive in the host, and cause disease. The database includes two virulence factor sequence datasets. Set A is the experimentally confirmed core dataset, whereas set B contains both the experimentally confirmed virulence factors in set A and predicted virulence factors. This database can help predict possible virulence factors in bacterial genomes that have not been studied in detail.

VFDB annotation results showed that the EPL 0201 strain contained the *vvhA*, *rtxA*, and *Wza* virulence factors (Table 3). This finding was consistent with the previously reported major virulence factors of *V. vulnificus* [47–49].

**Table 3:** VFDB annotation statistics

Gene	Gene ID	VFDB ID	Category	E-value	Score	Function
*vvhA*	GE003721	VF0611	Exotoxin	2.47E-189	612	Associated with enhanced growth in vivo and tissue necrosis in the small intestine
rtxA	GE004057	VF0265	Exotoxin	0E	4372	Induces cytopathic activities in host cells
*Wza*	GE002755	VF0465	Immune modulation	5.96E-160	457	Plays an important role in protecting bacteria from the host’s innate immune response

Note: “E-value” represents the expected value of the functional annotation results (the smaller the value, the more credible the results); “Score” represents the comprehensive score of the sequence alignment

#### CARD database annotations

CARD is a continuously updated database of information related to antibiotic resistance genes. It contains information describing antibiotics and their targets, including antibiotic resistance genes, related proteins, and mechanisms of antibiotic resistance. At the core of CARD is a highly developed Antibiotic Resistance Ontology for the classification of genetic data related to antibiotic resistance [39].

A search using the CARD database showed that the EPL 0201 strain contained *H-NS*, *TEM*, *sul1*, *tet*, *parE*, *APH*, and other resistance genes (Table 4). These resistance genes produce resistance to macrolides, penam, cephalosporin, carbapenems, aminoglycosides, tetracyclines, fluoroquinolones, sulfonamides, and rifampicin antibiotics.

**Table 4:** CARD database annotation statistics

Gene	Gene ID	ARO ID	Resistance	Resistance Mechanism
*H-NS*	GE001452	3,000,676	Penam, macrolide, cephalosporin, cephamycin, tetracycline, and fluoroquinolone	Antibiotic efflux
*CRP*	GE000225	3,000,518	Penam, macrolide, and fluoroquinolone	Antibiotic efflux
*adeF*	GE004010	3,000,777	Tetracycline and fluoroquinolone	Antibiotic efflux
*floR*	GE005069	3,002,705	Phenicol	Antibiotic efflux
*tet(59)*	GE005065	3,004,441	Tetracycline	Antibiotic efflux
*PBP3*	GE002506	3,004,446	Carbapenem, cephalosporin, monobactam, penam, and cephamycin	Antibiotic target alteration
*parE*	GE002526	3,003,316	Fluoroquinolone	Antibiotic target alteration
*mprF*	GE000717	3,003,324	Peptide	Antibiotic target alteration
*sul1*	GE005011	3,000,410	Sulfone and sulfonamide	Antibiotic target replacement
*sul2*	GE005074	3,000,412	Sulfone and sulfonamide	Antibiotic target replacement
*varG*	GE003041	3,004,289	Carbapenem	Antibiotic inactivation
*APH(6)-Id*	GE005072	3,002,660	Aminoglycoside	Antibiotic inactivation
*APH(3'')-Ib*	GE005073	3,002,639	Aminoglycoside	Antibiotic inactivation
*aadA16*	GE005009	3,002,616	Aminoglycoside	Antibiotic inactivation
*arr-3*	GE005007	3,002,848	Rifamycin	Antibiotic inactivation
*TEM-1*	GE005062	3,000,873	Penam, penem, cephalosporin, and monobactam	Antibiotic inactivation

*Note*: “ARO ID” represents the antibiotic resistance gene ID of the gene annotated into the database, “Resistance” represents the name of the antibiotic that caused the resistance, “Resistance Mechanism” represents the resistance mechanism of the resistance gene

### Antimicrobial susceptibility test

In total, 48 kinds of antibiotics were selected from 11 types of antibacterial drugs to conduct antibacterial drug susceptibility tests on *V. vulnificus* EPL 0201. The results showed that *V. vulnificus* EPL 0201 was sensitive to β-lactams such as imipenem, meropenem, piperacillin, and mezlocillin; macrolides such as azithromycin, erythromycin, roxithromycin, and midememycin; nitrofurans such as furazolidone; and aminoglycosides such as tobramycin (Table 5). It also showed varying degrees of resistance to different kinds of antibiotics, including β-lactams (ceftazolin, cefotaxime, ceftazidime, and cefoxitin), tetracyclines (tetracycline and doxycycline), sulfonamides (trimethoprim), quinolones (norfloxacin, ofloxacin, ciprofloxacin, and levofloxacin), aminoglycosides (kanamycin, spectinomycin, and neomycin), glycopeptides (teicoplanin and vancomycin), and lincosamides (lincomycin and clindamycin). These results indicate that *V. vulnificus* is a multi-drug resistant strain and confirmed the existence of its resistance genes.

**Table 5:** Antimicrobial susceptibility testing of the pathogenic strain EPL 0201

Group	Name	Drug concentration (μg per disk)	Bacteriostatic ring diameter (mm) ∗	Sensitivity†
β-Lactams	Cefazolin	30	17.66	R
	Cephalothin	30	15.98	I
	Cefotaxime	30	15.43	R
	Ceftriaxone	30	31.02	S
	Ceftazidime	30	20.26	I
	Cefoperazone	75	26.74	S
	Cefepime	30	21.26	S
	Cefoxitin	30	15.68	R
	Amoxicillin	10	11.52	R
	Azlocillin	75	16.36	R
	Piperacillin	100	21.28	S
	Mezlocillin	75	20.64	S
	Ticarcillin	75	23.26	S
	Ampicillin	10	10.16	R
	Penicillin	10	8.66	R
	Imipenem	10	29.72	S
	Meropenem	10	31.72	S
Aminoglycosides	Kanamycin	30	17	I
	Spectinomycin	100	15.26	I
	Neomycin	30	19.12	I
	Streptomycin	10	6.35	R
	Amikacin	30	13.88	R
	Gentamicin	10	16.71	S
	Tobramycin	10	15.64	S
Glycopeptides	Teicoplanin	30	8.67	R
	Vancomycin	30	10.65	R
Quinolones	Nalidixic acid	30	36.51	S
	Ciprofloxacin	5	18.68	I
	Levofloxacin	5	25.03	S
	Enrofloxacin	5	34.9	S
	Norfloxacin	10	11.66	R
	Ofloxacin	5	15.54	I
Chloramphenicols	Chloramphenicol	30	16.98	I
Macrolides	Azithromycin	15	31.37	S
	Erythromycin	15	29.06	S
	Roxithromycin	15	23.01	S
	Midecamycin	15	24.43	S
	Claricid	15	23.16	S
Nitrofurans	Furazolidone	300	21.5	S
Lincosamides	Lincomycin	2	10.02	R
	Clindamycin	2	13.96	I
Sulfonamides	Trimethoprim	23.75	6.35	R
Tetracyclines	Tetracycline	30	15.04	I
	Doxycycline	30	18.86	I
	Minocycline	30	32.77	S
Others	Rifampin	5	11.98	R
	Methoxyamine	5	6.35	R
	Polymyxin B	300	11.03	I

*Note*: “blank” means no inhibition zone, “S” means highly sensitive, “I” means moderately sensitive; and “R” means resistant

## Discussion

### Isolation and identification of the EPL 0201 strain

In this study, the EPL 0201 strain was isolated from the kidney, spleen, and intestine of diseased gentian groupers. The 16S rRNA gene sequence is the genetic marker most commonly used for bacterial taxonomy [50]; however, owing to the extremely high similarity of the 16S rRNA gene sequences in *Vibrio* species, the accuracy of identification at the species level is limited [51]. *V. vulnificus* biotypes are complex, and routine identification of 16S rRNA can lead to erroneous results [43]. Therefore, identification was performed using the gyrB gene, the API 20E identification system, and the virulence gene. The gyrB gene is variable and conservative, owing to its genetic codon usage that allows the DNA sequence to undergo relatively many substitutions without changing the amino acid sequence [52]. Therefore, this gene is valuable for the discrimination of *Vibrio spp*.

The 16S rRNA gene and gyrB sequences of the EPL 0201 strain were analyzed using nucleotide BLAST against the NCBI database. More than 99% homology with the sequence of the standard strain of *V. vulnificus* was observed. A phylogenetic tree was also constructed, and the results showed that the EPL 0201 strain clustered with *V. vulnificus*. Several reports on *V. vulnificus* have indicated that the API 20E system is effective for identifying *V. vulnificus*, with 85–99% accuracy [42, 43]. The code of the EPL 0201 strain, according to the API 20E coding manual is 500600557, which is consistent with the physiological and biochemical characteristics of *V. vulnificus* strain ATCC 33,149. In order to improve the accuracy and reliability of the identification, and to avoid false negative results caused by API 20E in the identification of trauma biotypes 1 and 2, resulting in identification errors, additional experiments with NaCl peptone water supplementation were performed, based on the study by Biosca et al. [43]. The results were as expected and the EPL 0201 strain was identified as *V. vulnificus* biotype 2. The presence of several virulence genes in the EPL 0201 strain indicated that the synergistic effects conferred by combinations of these genes contribute to its pathogenicity. This result is in accord with the observation that combinations of virulence genes are commonly present in particularly virulent strains of *V. vulnificus* [27]. According to research by Shao et al. [30], a bacterial strain can be considered highly virulent if its LD_50_ values are within the range of 10^5^–10^6^ CFU g^−1^ fish body weight. In our study, the LD_50_ of the EPL 0201 strain was calculated as 1.097 × 10^5^ CFU g^−1^, indicating that it is highly virulent to the pearl gentian grouper. Fish infected with *V. vulnificus* tended to exhibit various clinical symptoms, including skin lesions, water accumulation in their abdomens, and diseased and necrotic tissues and organs. Different *V. vulnificus* strains sometimes, but not always, cause similar clinical symptoms. Even the same *V. vulnificus* could cause divergent clinical symptoms in different fish. In this study, pearl gentian grouper infected with the EPL 0201 strain developed abdominal effusions. However, in related studies, this symptom was not present after infection with *V. vulnificus*[44, 53, 54]. The results showed that the pearl gentian grouper infected with *V. vulnificus* EPL 0201 strain developed skin lesions. This is the same as the findings of Thawonsuwan et al. [44]. However, in the study by Fouz and Li et al. [53, 54], this symptom did not manifest itself after infection with a *V. vulnificus* strain.

### Whole-genome sequencing and annotation of the EPL 0201 strain

*V. vulnificus* causes a large number of deaths in marine aquaculture fish and seriously threatens the development of aquaculture. It can also infect humans and other mammals, thereby impacting public health. Advances in bioinformatics and genome sequencing have facilitated the understanding of microbial diversity, evolution, and interspecies interactions [55]. Whole-genome sequencing technology has been widely used in the identification and analysis of pathogenic bacteria affecting animals and plants and can aid the systematic study of the evolution, pathogenic mechanisms, and interaction mechanisms of pathogenic bacteria at the molecular level. It provides valuable reference data for the identification and classification of pathogenic bacteria, the detection of drug resistance, the formulation of disease prevention strategies, and the development of vaccines [56, 57].

In recent years, there have been increasing numbers of reports on *V. vulnificus*, but there are few reports describing the isolation of this bacterium from the pearl gentian grouper, and research on the pathogenesis of this bacterium is still incomplete [11, 44]. In this study, *V. vulnificus* was isolated from diseased pearl gentian grouper and systematically analyzed. The analysis of the genome sequence of EPL 0201 can provide a theoretical basis for the in-depth study of its mechanisms of pathogenesis. In this study, the whole-genome sequence of *V. vulnificus* EPL0201 was determined, and the genome (5,769,851 bp) was assembled into three chromosomes and two plasmids. EPL 0201 had a larger genome than that of the reference strain for this bacterium, from the JGI IMG/MER database. It has been reported that the larger the genome, the more likely it is to contain metabolism-related and resistance genes, providing a unique mechanism for adaptation to different living environments [58]. VFDB annotations confirmed that the strain carried three virulence factor genes: *vvhA*, *rtxA*, and *Wza*. Related studies have shown that these genes are closely related to bacterial pathogenicity [29, 59]. The virulence genes were identified via different strains is not completely consistent between studies; thus, virulence genes should be studied in greater depth [58]. KEGG database annotation found that this strain has abundant, complete metabolic pathways. The presence of multiple metabolic pathways enable organisms to continuously exchange substances and energy, maintain metabolism, and respond to the external environment in a timely manner, thereby enhancing the environmental adaptability of strains [60].

### Drug resistance analysis of V. vulnificus EPL 0201

Aquatic bacteria, which are able to adapt to their environment, usually contain several antibiotic resistance factors, which enable them to resist antibiotics that may be present in aquaculture ecosystems [61]. Analysis using the CARD database revealed that the EPL 0201 strain had resistance genes such as *TEM*, *sul1*, *tet*, *parE*, and *APH*, which can confer resistance to β-lactams (carbapenems), aminoglycosides, tetracyclines, fluoroquinolones, sulfonamides, and rifampicin. This result suggests that the isolate is a multi-drug resistant strain. According to the results of the drug susceptibility test, the EPL 0201 strain was resistant to antibiotics such as β-lactams (cephalosporins), aminoglycosides, tetracyclines, quinolones, sulfonamides, glycopeptides, and lincosamides. These results showed that this strain was multi-drug resistant *V. vulnificus* and confirmed the resistant genes identified in its genome. However, we found that the strain was highly sensitive to carbapenem and macrolides, a finding which may be related to the type and frequency of carbapenem antibiotics used in this area or to the weakening and loss of function caused by the mutation of resistance genes [23, 62]. Studies have also reported that environmental factors may mediate the mutation or methylation of bacterial resistance genes, thereby changing their resistance [63]. These results also indicate that some drug resistance phenotypes are not completely consistent with resistance gene carriers. Interestingly, the EPL 0201 strain showed varying degrees of resistance to 13 of the 22 antimicrobial agents recommended by the Centers for Disease Control and Prevention for the treatment of *Vibrio spp.* infection [64], indicating that this strain has gradually become insensitive to the quick-acting drugs used for the treatment of *V. vulnificus* infection, which should be a cause for alarm.

## Supplementary Information

**Additional file 1: Table F1.** Primers used in this study. **Table F2.** Raw sequencing data. **Table F3.** Filtered sequencing data. **Table F4.** Summarized data of gene prediction. **Table F5.** Assembly index. **Fig. S1.** Electrophoresis of the DNA isolated from the EPL 0201 strain. **Fig. S2.** Sequencing data length distribution of the EPL 0201 train. **Fig. S3.** Function classification statistics of eggNOG functional genes. Note: The abscissa represents the content of each eggNOG classification, and the ordinate represent the relative content of the number of corresponding functional genes. **Fig. S4.** GO function annotation clasification statistics chart. Note: The abscissa represents the content of each GO category, the left of the ordinate represents the percentage of genes, and the right of the ordinate represents the numbers of genes. **Fig. S5.** KEGG annotation classification statistics. Note: The ordinate represents the KEGG secondary classification, and the abscissa represents the percentage. **Fig. S6.** Original agarose gel electropherogram. **Fig. S7**. pictures of naturally occurring fish. **Fig. S8.** Electrophoresis of the DNA isolated from teh EPL 0201 strain (original image).

## Data Availability

The 16S rRNA gene sequence of EPL 0201 strain has been submitted to GenBank (https://www.ncbi.nlm.nih.gov/genbank/; accession number OL687343).
