# Supplementary material for: Identification and whole-genome sequencing analysis of Vibrio vulnificus strains causing pearl gentian grouper disease in China
Source: BMC Microbiol. 2022 Aug 16;22:200. doi: 10.1186/s12866-022-02610-1 (PMC9380395; doi:10.1186/s12866-022-02610-1)
Supplement: Supplementary file 1 — Additional file 1: Table F1. Primers used in this study. Table F2. Raw sequencing data. Table F3. Filtered sequencing data. Table F4. Summarized data of gene prediction. Table F5. Assembly index. Fig. S1. Electrophoresis of the DNA isolated from the EPL 0201 strain. Fig. S2. Sequencing data length distribution of the EPL 0201 train. Fig. S3. Function classification statistics of eggNOG functional genes. Note: The abscissa represents the content of each eggNOG classification, and the ordinate represent the relative content of the number of corresponding functional genes. Fig. S4. GO function annotation clasification statistics chart. Note: The abscissa represents the content of each GO category, the left of the ordinate represents the percentage of genes, and the right of the ordinate represents the numbers of genes. Fig. S5. KEGG annotation classification statistics. Note: The ordinate represents the KEGG secondary classification, and the abscissa represents the percentage. Fig. S6. Original agarose gel electropherogram. Fig. S7. pictures of naturally occurring fish. Fig. S8. Electrophoresis of the DNA isolated from teh EPL 0201 strain (original image). [file 12866_2022_2610_MOESM1_ESM.pdf]

Appendix

Table F1. Primers used in this study

**Genes**	**Primers**	**Primer sequence (5′-3′)**	**Reference**
16S rRNA	16S rRNA-F	AGAGTTTGATCCTGGCTCAG	Weisburg et al. (1991)
	16S rRNA-R	ACGGCTACCTTGTTACGACTT	
gyrB	gyrB-F	ATCATGACGGTACTGCAYGC	Sawabe et al. (2007)
	gyrB-R	ACGTCTGCGTCGGTCATGAT	
vvgC	vvgC-F	CATGAT AGCTTCGGCTCA A	Warner and Oliver (2008)
	vvgC-R	CACTACCACCTTCCTCACGAC	
rtxA	rtxA-F	CGGGATCCTATGGCGTGAACGGCGAAG	Amaro et al. (2015)
	rtxA-R	CGGGATCCAGCAGCCACAAGCGATTC	
vvhA	vvhA-F	TTCCAACTTCAAACCGAACTATGA	Rivera et al. (2001)
	vvhA-R	ATTCCAGTCGATGCGAATACGTTG	
Wza	Wza-F	ATTCCGTGACCGATTGAGCGT	Rivera et al. (2001)
	Wza-R	GCAGTAGAAGATACACCTAGG	
OmpU	OmpU-F	ACGCTGACGGAATCAACCAAAG	Amaro et al. (2015)
	OmpU-R	GCGGAAGTTTGGCTTGAAGTAG	

Table F2. Raw sequencing data

SamID	SeqNum	SumBase	N50Len	MeanLen	MaxLen	Meanqual	(C + G)%
EPL0201	56658	429195689	8443	26624	7575	51	44.96

Note: “SeqNum” refers to sequence number, “SumBase” and “SeqNum” refer to the total number of bases, and “N50Len” means data N50 length (bp).

Table F3. Filtered sequencing data

SamID	SeqNum	SumBase	N50Len	MeanLen	MaxLen	Meanqual	(C + G)%
EPL0201	56352	428664473	8447	7606	26624	50.87	44.96

Note: “SeqNum” refers to sequence number, “SumBase” and “SeqNum” refer to the total number of bases, and “N50Len” means data N50 length (bp).

Table F4. Summarized data of gene prediction

SamID	CotNum	CotLen	N50Len	(C + G)%	Geneset	rRNA	tRNA	Other ncRNA
EPL0201	5	5769851	3330901	46.41	5194	34	121	72

Note: “CotNum” means Contig Number, “CotLen” means Contig Length (bp), “N50Len” means Contig N50 length (bp), and “Geneset” means Geneset number (Quality standards < 8000).

Table F5. Assembly index

**Contig ID**	**Length**	**Type**	**Topology**
Contig00001	3330901	chromosome	circular
Contig00002	1952706	chromosome	circular
Contig00003	429477	plasmid	circular
Contig00004	42131	plasmid	linear
Contig00005	14636	plasmid	linear

Note: Contig_ID: contig number; Type: Contig type (chromosome: the coverage of the plasmid sequence aligned to the database is >80; plasmid: the coverage of the plasmid sequence aligned to the database is <80); Circular: Whether the contig is circular.

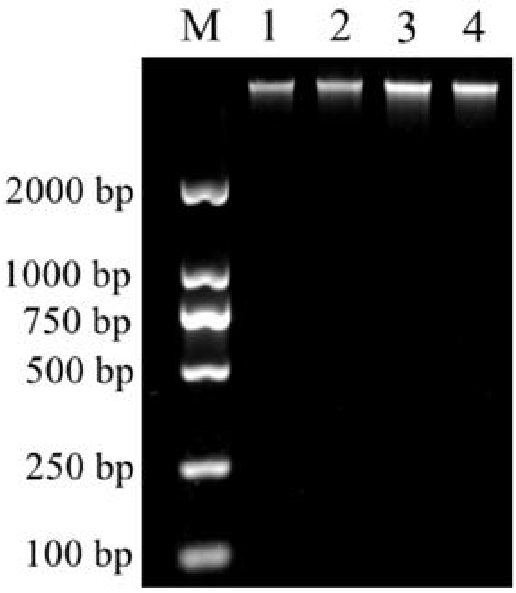

Fig. S1. Electrophoresis of the DNA isolated from the EPL 0201 strain.

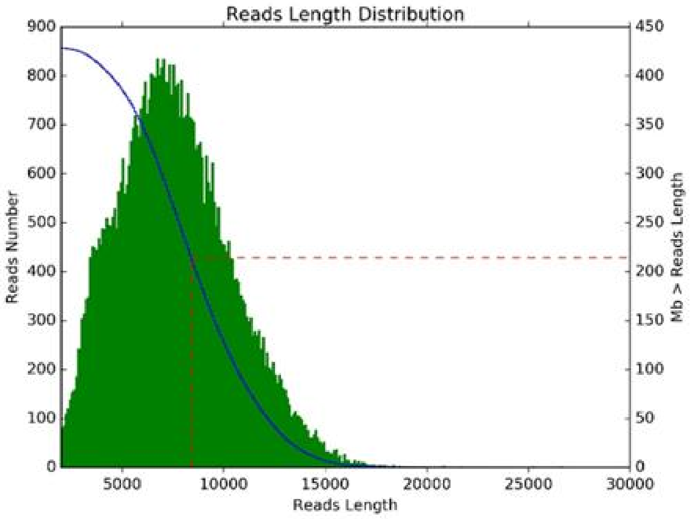

Fig. S2. Sequencing data length distribution of the EPL 0201 strain

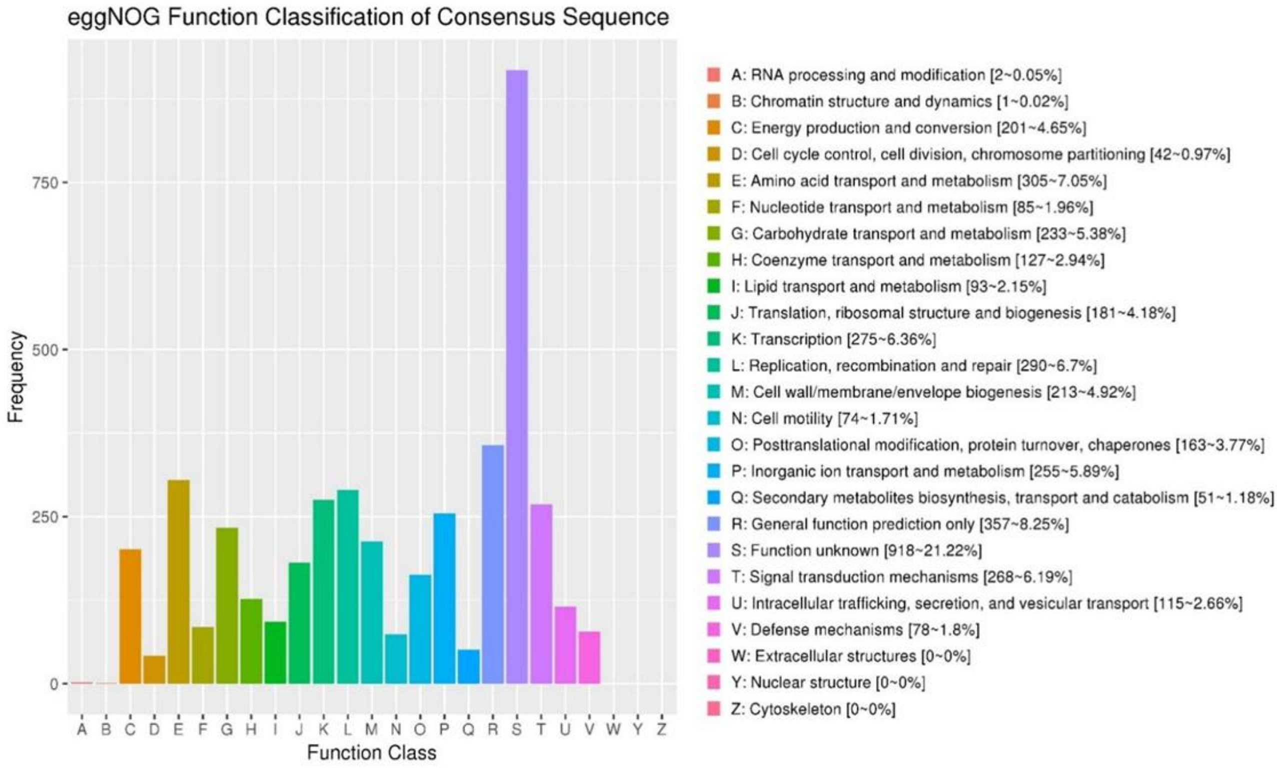

Fig. S3. Function classification statistics of eggNOG functional genes. Note: The abscissa represents the content of each eggNOG classification, and the ordinate represents the relative content of the number of corresponding functional genes.

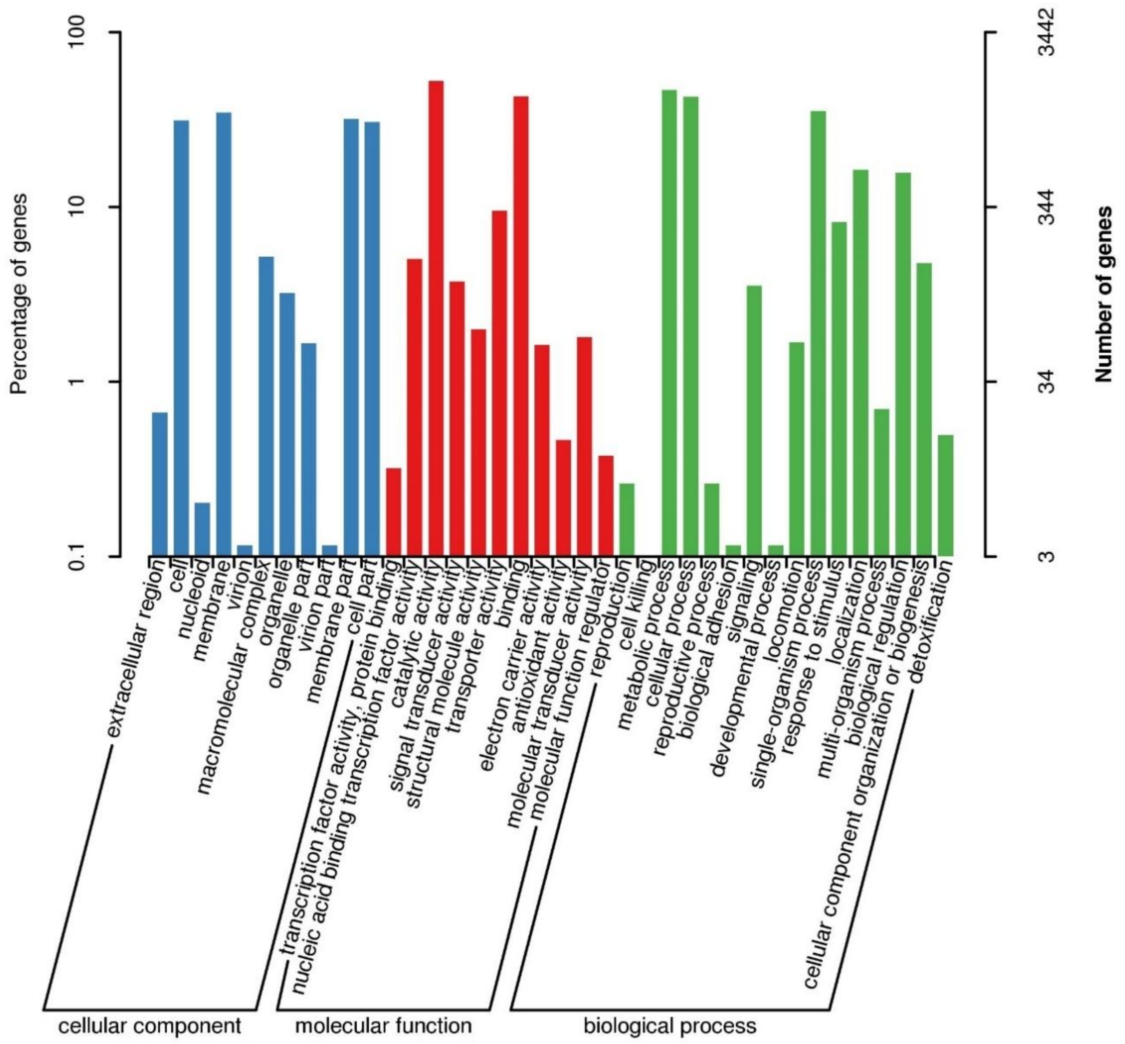

Fig. S4. GO function annotation classification statistics chart. Note: The abscissa represents the content of each GO category, the left of the ordinate represents the percentage of genes, and the right of the ordinate represents the number of genes.

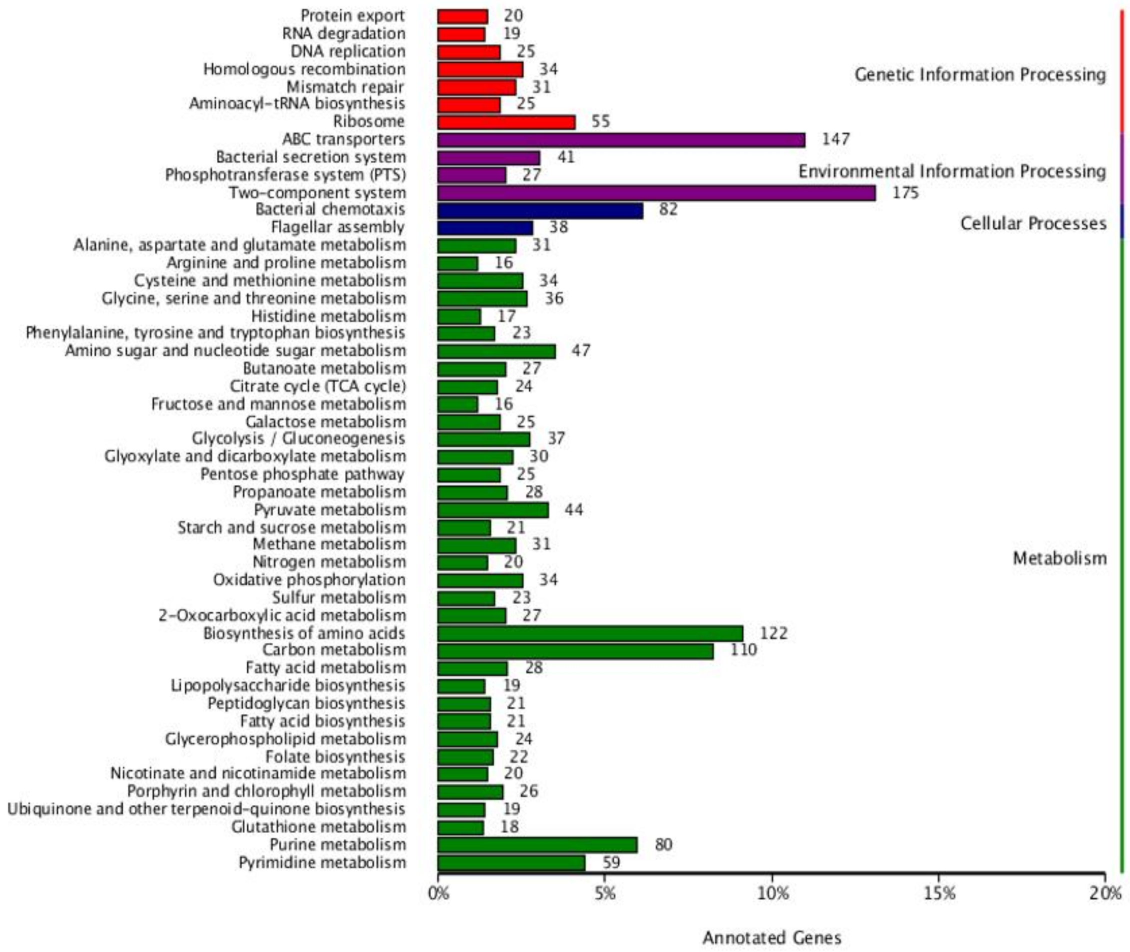

Fig. S5. KEGG annotation classification statistics. Note: The ordinate represents the KEGG secondary classification, and the abscissa represents the percentage.

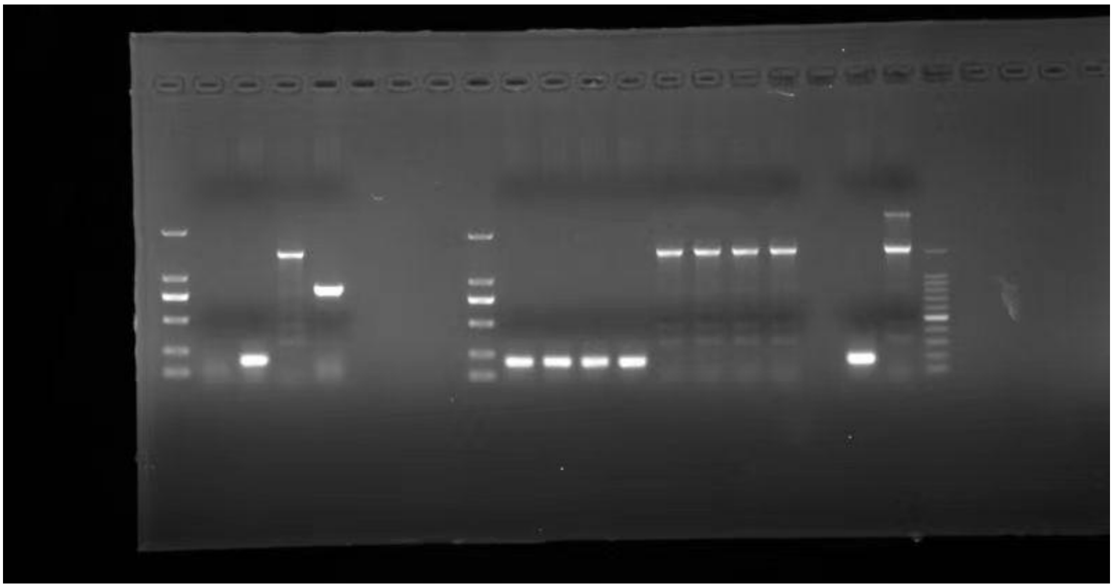

Fig. S6. Original agarose gel electropherogram.

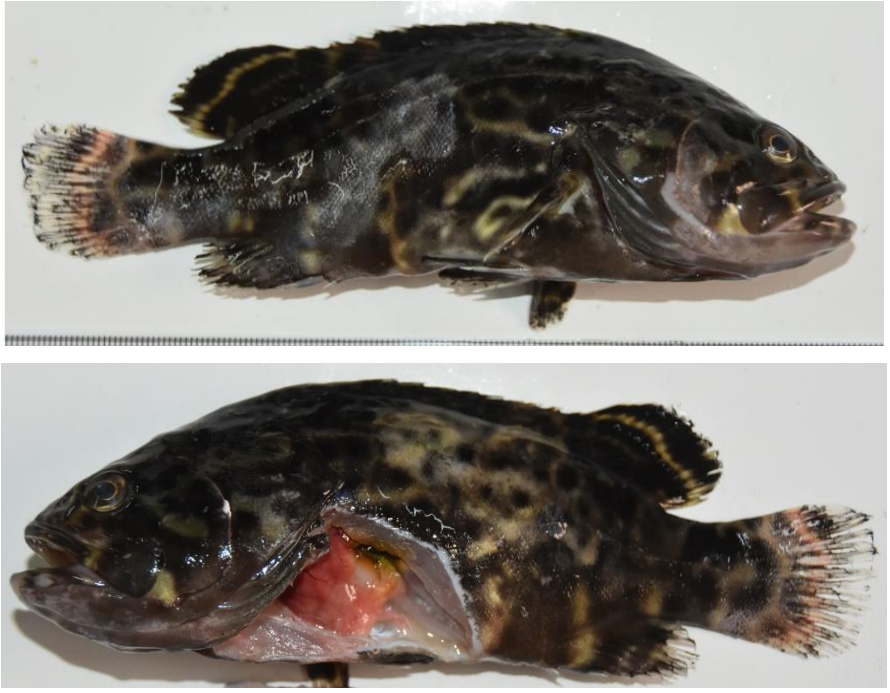

Fig. S7. pictures of naturally occurring fish

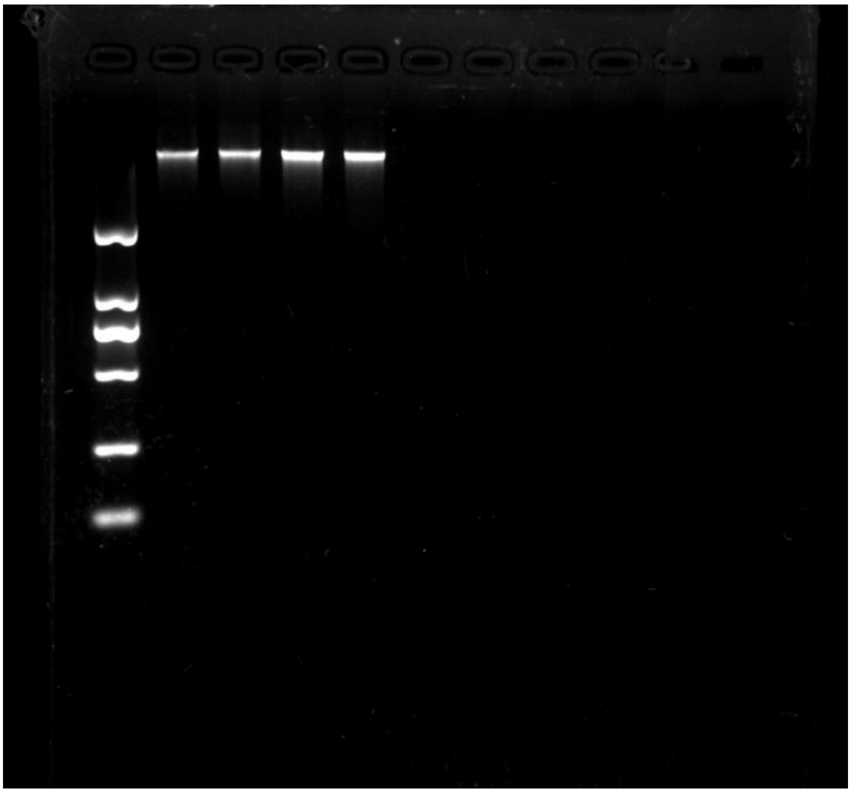

Fig. S8. Electrophoresis of the DNA isolated from the EPL 0201 strain（original image）
